# Comparative Study on the Characteristics of *Weissella cibaria* CMU and Probiotic Strains for Oral Care

**DOI:** 10.3390/molecules21121752

**Published:** 2016-12-20

**Authors:** Hye-Jin Jang, Mi-Sun Kang, Sung-Hun Yi, Ji-Young Hong, Sang-Pil Hong

**Affiliations:** 1 Division of Strategic Food Research, Korea Food Research Institute (KFRI), 1201-62 Anyangpangyo-ro, Bundang-gu, Seongnam-si, Gyeonggi-do 13539, Korea; Jang.Hye-jin@kfri.re.kr (H.-J.J.); sunghunyi@kfri.re.kr (S.-H.Y.); Hong.Ji-young@kfri.re.kr (J.-Y.H.); 2 Oradentics Co., Ltd., 3F KM Building 23, Teheran-ro 77-gil, Gangnam-gu, Seoul 06157, Korea; jieenkang@oradentics.com

**Keywords:** *Weissella cibaria*, probiotics, oral care, hydrogen peroxide, biofilm, antibacterial, volatile sulfur compound

## Abstract

Probiotics have been demonstrated as a new paradigm to substitute antibiotic treatment for dental caries, gingivitis, and chronic periodontitis. The present work was conducted to compare the characteristics of oral care probiotics: *Weissella cibaria* CMU (Chonnam Medical University) and four commercial probiotic strains. Survival rates under poor oral conditions, acid production, hydrogen peroxide production, as well as inhibition of biofilm formation, coaggregation, antibacterial activity, and inhibition of volatile sulfur compounds were evaluated. The viability of *W. cibaria* CMU was not affected by treatment of 100 mg/L lysozyme for 90 min and 1 mM hydrogen peroxide for 6 h. Interestingly, *W. cibaria* produced less acid and more hydrogen peroxide than the other four probiotics. *W. cibaria* inhibited biofilm formation by *Streptococcus mutans* at lower concentrations (*S. mutans*/CMU = 8) and efficiently coaggregated with *Fusobacterium nucleatum*. *W. cibaria* CMU and two commercial probiotics, including *Lactobacillus salivarius* and *Lactobacillus reuteri*, showed high antibacterial activities (>97%) against cariogens (*S. mutans* and *Streptococcus sobrinus*), and against periodontopathogens (*F. nucleatum* and *Porphyromonas gingivalis*). All of the lactic acid bacterial strains in this study significantly reduced levels of hydrogen sulfide and methyl mercaptan produced by *F. nucleatum* and *P. gingivalis* (*p* < 0.05). These results suggest that *W. cibaria* CMU is applicable as an oral care probiotic.

## 1. Introduction

More than 700 bacterial species have been detected in the oral cavity [1], and the balance among these bacteria regulates the development of oral diseases such as oral caries, gingivitis and chronic periodontitis [2]. It is generally accepted that the oral microbiota, along with host and diet factors, influences the development of dental caries [3,4]. *Streptococcus mutans* and *Streptococcus sobrinus* are example of oral bacteria involved in the early stage of dental caries [5]. The development of dental caries involves of secretion of GTF (glucosyltranferase) by *S. mutans*, which produces sticky, extracellular dextran-based polysaccharides that allow the bacteria to cohere, forming plaque that causes dental caries [6]. Periodontitis has been associated with the decrease in quality of life due to impairment of masticatory function and attractiveness [7], and has also been implicated in several systematic diseases such as cerebrovascular disease, cardiovascular disease, diabetes and aspiration pneumonia [8,9]. The cause of periodontitis is related to bacterial plaques and metabolites, which are produced by Gram-negative bacteria, such as *Porphyromonas gingivalis* and *Fusobacterium nucleatum*, in the oral cavity [10].

A number of studies on the use of probiotics for the improvement of dental caries, gingivitis, and chronic periodontitis have created a new paradigm for the substitution of existing antibiotic treatments [11,12,13,14,15]. According to the definition by FAO/WHO, probiotics are “live microorganisms, which when administered in adequate amounts, confers a health benefit to the host” [16]. Lactic acid bacteria and *Bifidobacteria* are the most common types of microbes used as probiotics [16]. Medical conditions that have the potential to be treated with probiotics include diarrhea, gastroenteritis, irritable bowel syndrome, inflammatory bowel disease (Crohn’s disease and ulcerative colitis), cancer, depressed immune function, inadequate lactase digestion, infant allergies, failure-to-thrive, hyperlipidemia, hepatic diseases, *Helicobacter pylori* infection, genitourinary tract infections, and others [17,18,19]. Regarding studies on probiotics in oral care, *Lactobacillus reuteri* [11] and *Lactobacillus salivarius* [15] were reported to prevent dental caries and periodontitis. Recently, *Weissella cibaria* CMS1 was shown to have preventive effects on biofilm formation [13] and on production of the main sulfur compound in halitosis [12]. Additionally, *W. cibaria* exhibited antibacterial activity against periodontitis bacteria [12], suggesting its use as a probiotic in oral care products. *Weissella* is a Gram-positive bacteria within the family *Leuconostocaceae* [20]. *W. cibaria* is a lactic acid bacteria that is rod-shaped, non-spore forming, and is heterofermentative, using sugar as a substrate. The morphology of *Weissella* species varies from spherical or lenticular cells to irregular rods. These species are widely found in saliva or in fermented foods, such as kimchi [21].

In this study, the characteristics of *W. cibaria* CMU (US 7250162B2), were compared with those of probiotic strains from commercial oral care products based on in vitro analysis, such as antibacterial activity and inhibition of biofilm and sulfur compound formation in addition to other basic analysis.

## 2. Results

### 2.1. Resistance against Lysozyme and Hydrogen Peroxide

#### 2.1.1. Lysozyme

**Figure 1:**
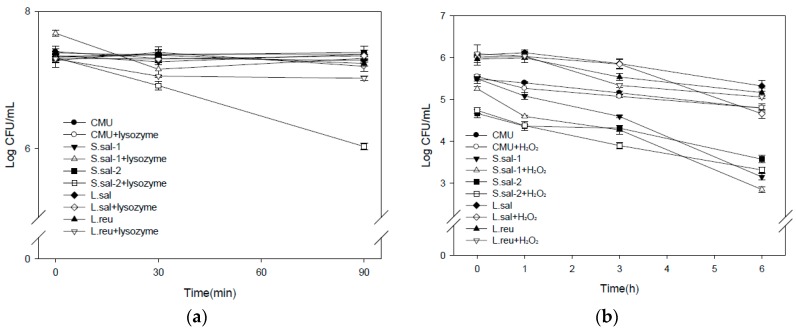
The survival level of oral probiotic strains after treatment with (**a**) 0.01% lysozyme (*w*/*v*), and (**b**) 1 mM hydrogen peroxide.

Lactic acid bacteria were treated with 100 mg/L lysozyme solution from 30 to 90 min as a means of establishing poor survival conditions. Among lactic acid bacteria, the survival ratios of *W. cibaria* CMU, L. sal and S. sal-1 were not affected by 90 min lysozyme treatment, while S. sal-2 and *L. reuteri* were reduced by 18.5% and 3.0% at 90 min, respectively (Figure 1a).

#### 2.1.2. Hydrogen Peroxide

The survival level for lactic acid bacteria treated with 1 mM H_2_O_2_ for 1, 3, and 6 h were assessed. The survival of *W. cibaria* CMU and L. reu was not significantly inhibited by hydrogen peroxide. However, S. sal-1, S. sal-2, and L. sal showed a viability ratio of 90.49%, 92.83%, and 87.56% at 6 h, respectively (Figure 1b).

### 2.2. Acidogenic Potential

The acidogenic potential (production of acid value, PAV) of the probiotic strains ranged from 22.28 to 56.47. In particular, *W. cibaria* CMU showed the highest PAV in carbohydrates: glucose 37.29, fructose 40.09, sucrose 47.28, lactose 41.71, and yeast extract 56.31 (Table 1).

### 2.3. Hydrogen Peroxide Production Potential

**Figure 2:**
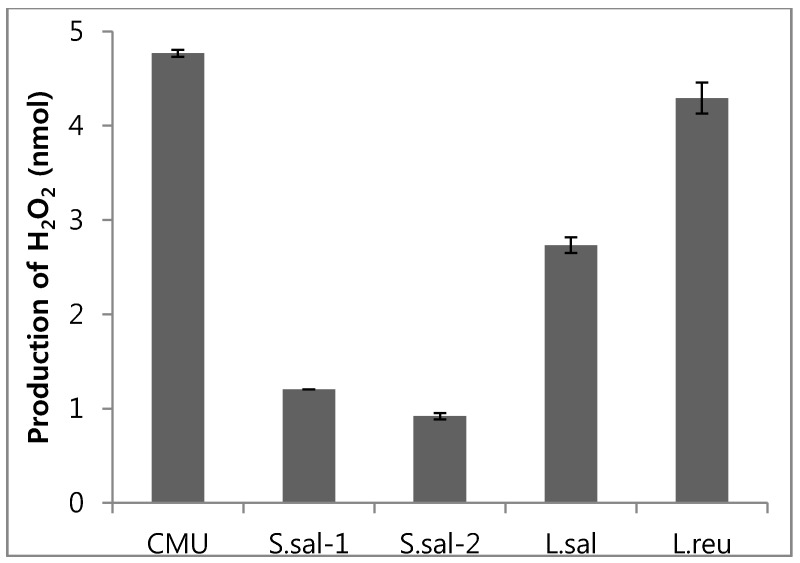
Hydrogen peroxide production activity of probiotics.

Among lactic acid bacteria in this study, *W. cibaria* CMU produced the most hydrogen peroxide, followed by L. reu, L. sal, S. sal-1, and S. sal-2 (Figure 2).

### 2.4. Inhibition of Biofilm Formation

As shown in Table 2, *W. cibaria* CMU and L. sal strongly inhibited biofilm formation in the mixed culture of *S. mutans* by 94.7% ± 0.3% and 93.9% ± 0.5%, respectively, followed by L. reu (85.3% ± 4.0%), and S. sal-1 (59.5% ± 2.8%) at a 1:1 ratio. In the case of S. sal-2, there was no significant inhibition of biofilm formation (Table 2). In the dose-dependency assay, *W. cibaria* CMU showed more than 95% inhibition through the entire dose range (2:1, 4:1, and 8:1), whereas S. sal-1 and L. sal showed dose-dependent inhibition. Furthermore, S. sal-2 and L. reu did not show any inhibition across the range of doses.

### 2.5. Antibacterial Activity

When assessing the antibacterial activity of the probiotics on dental caries bacteria (Table 3) or on periodontal bacteria (Table 4), *W. cibaria* CMU, L. sal, and L. reu showed higher antibacterial activity as indicated by more than 97% growth inhibition when compared to the other probiotic strains at a 1:1 ratio. Additionally, L. sal maintained a similar level of antibacterial activity at a 4:1 ratio, except for *S. mutans*. Also, *W. cibaria* CMU, L. sal, and L. reu showed antibacterial activity against *F. nucleatum* and *P. gingivalis*, as indicated by more than 95% growth inhibition at a 1:1 ratio. In particular, antibacterial activities of *W. cibaria* CMU, L. sal, and L. reu against *P. gingivalis* were shown to be more than 98% at an 8:1 ratio. However, S. sal-1 and S. sal-2 did not have antibacterial activity against dental caries or periodontal bacteria as compared to the other lactic acid bacteria.

### 2.6. Coaggregation

There was no significant coaggregation between the probiotics and dental caries bacteria or periodontal bacteria. Among probiotics, *W. cibaria* CMU showed the highest coaggregation with *F. nucleatum* (81.2%), followed by S. sal-1 (78.9%), S. sal-2 (72.7%), and L. reu (49.6%), respectively (Table 5).

### 2.7. Inhibition of VSC Production

When assay the inhibition of VSC production by the lactic acid bacteria, *W. cibaria* CMU showed the strongest inhibition as compared to the other lactic acid bacteria. *W. cibaria* CMU inhibited H_2_S and CH_3_SH production by *F. nucleatum* by 97.0%, and by *P. gingivalis* by 93.9% (Table 6).

## 3. Discussion

Dental plaque-related diseases (cavities, gingivitis, and periodontitis) has been traditionally controlled by mechanical non-specific removal of plaques. However, a number of novel treatment approaches aim to inhibit the growth of pathogenic bacteria or to remove their toxins.

Recently, antibacterial plant-originated substances [22] or probiotics have been applied as new tools for the improvement of dental health. They have been used to substitute existing antibiotic treatments due to increased resistant bacteria [11,12,13,14,15]. Probiotics not only have antibacterial activity, but they also have inhibitory effects on the reappearance of oral pathogenic bacteria. When choosing the best probiotics for oral health care, it is important to screen probiotics for their viability under poor oral conditions, ability to lower acid production, antibacterial activity, inhibition of biofilm formation, and for oral malodour.

Within saliva, there exists lysozyme and hydrogen peroxide. Lysozyme has an enzymatic activity that cleaves the 1,4-linkage between *N*-acetylmuramic acid and *N*-acetylglucosamine in the peptidoglycan in bacterial cell wall [23]. Hydrogen peroxide produces hydroxyl radicals that inhibit the growth of pathogenic bacteria. Additionally, hydroxyl radicals can react with nucleic acids to cause damage to genes, and can also increase permeability, limit membrane transportation and denature proteins in cells [24]. Therefore, the resistance capability of lactic acid bacteria to lysozyme or hydrogen peroxide treatment can be used to predict viability in oral conditions.

In the assay for lysozyme resistance, the viabilities of *W. cibaria* CMU and L. sal were not affected by treatment with 100 mg/L lysozyme for 90 min. Furthermore, *W. cibaria* CMU and L. reu showed higher resistance with 1 mM hydrogen peroxide treatment, suggesting that *W. cibaria* CMU is viable under poor oral conditions.

It is known that high lactic acid producing bacteria are not good for oral health because they may cause dental caries [6]. From the calculation of PAV, in which higher values mean lower bacterial acid forming ability, the induction ratio of dental caries from *W. cibaria* CMU was expected to be lower than that of the other lactic acid bacteria in this study. Moreover, lactic acid bacteria can produce organic acids, CO_2_, diacetyl, lower molecule antimicrobial materials, bacteriocins, and anticohesive materials [17]. Among these products, it is reported that hydrogen peroxide, a representative antibacterial material, induces changes in the bacterial community of the oral cavity and inhibits growth of *F. nucleatum* that causes oral malodor [12]. *W. cibaria* CMU has shown higher hydrogen peroxide forming ability, as compared with the other lactic acid bacteria. Therefore, it was suggested that *W. cibaria* CMU may have a good ability to reduce oral malodour.

Caries are due to the accumulation of dental plaque (a microbial biofilm) on the tooth surface and at the gingival margin, the vast of majority of which is composed of bacteria [3,4]. As a strategy for the prevention of caries caused by microbial biofilms from *S. mutans*, probiotics should compete with biofilm forming bacteria and inhibit their growth.

Insoluble glucan is the principal constituent of oral biofilm, and also constitutes a potential site for the formation of carious lesions. The production of glucans from sucrose by GTF is one of the mechanisms underlying the virulence of *S. mutans* [6]. Therefore, the effective suppression of insoluble glucan formation may constitute a viable approach to the prevention of biofilm induced oral diseases such as dental caries.

In this work, *W. cibaria* CMU and L. sal strongly inhibited biofilm formation by *S. mutans*, and *W. cibaria* CMU also showed more than 95% inhibition across all doses of *S. mutans* used. This results suggest that *W. cibaria* CMU exhibits functions well as a probiotic. S. sal-2 was previously reported to form BLIS (bacteriocin-like inhibitory substances), an antibacterial material and dextranase, that results in the prevention of the accumulation of dental plaque [25]. However, there was no inhibitory activity against biofilm formation in this study. *W. cibaria* CMU, L. sal and L. reu also exhibited antibacterial activity for caries and periodontal bacteria similar to previous reports [11,12,15]. Contrary to other studies [14,25], S. sal-1 and S. sal-2 did not exhibit good antibacterial activity. In addition, in the present study, *W. cibaria* CMU at dose range (*S. mutans*:CMU = 8:1) strongly inhibited *S. mutans* biofilm formation by 95.4% ± 0.1%. This result was supported by the report of previous study [13] that dextran (water-soluble glucan) from *W. cibaria* inhibited the synthesis of water-insoluble glucan by *S. mutans*, via the conversion of GTF activity from the production of water-insoluble glucan to the production of water-soluble glucan.

Several *Weissella*, *Leuconostoc*, *Streptococcus*, and *Lactobacillus* spp. can produce dextran. Dextran primarily comprises α-1,6-d-glucan and is synthesized by dextransucrase. It is known that dextran can be used as a potential prebiotic for health benefits owing to stimulating the growth of probiotic bacteria such as *Bifidobacterium* spp. and *Lactobacillus acidophilus* [26].

In addition, a number of studies have reported that probiotics inhibit a variety of bacteria including *P. gingivalis*, *Treponema denticola*, *Aggregatibacter actinomycestemcomitans*, and *Tannerella forsythia* [27,28]. *F. nucleatum* is found in the oral cavity, and can serve as a bridge organism, via cohesion and coaggregation, for other bacteria and assist in the inhabitation of the oral cavity [29]. The viability of *F. nucleatum* is advantageous in the oral cavity as it cannot be easily removed by saliva. Therefore, coaggregation with lactic acid bacteria has been suggested to help remove pathogenic bacteria and prevent plaque formation.

In the evaluation of coaggregation between 5 lactic acid bacteria with two dental caries, *F. nucleatum* and *P. gingivalis*, and two periodontal bacteria, *S. mutans* and *S. sobrinus*, *F. nucleatum*, *W. cibaria* CMU showed the highest coaggregation with *F. nucleatum*, followed by S. sal-1, S. sal-2 and L. reu, but L. sal did not show any coaggregation (Table 5). The above results using *W. cibaria* CMU are in good agreement with a previous study [12].

The benefits of using probiotics for halitosis, oral malodor, have been demonstrated. The main compounds related to halitosis are volatile sulfur compounds produced by Gram-negative bacteria such as *F. nucleatum*, and *P. gingivalis.* These volatile sulfur compounds include hydrogen sulfide and methyl mercaptan, both of which comprise about 90% of the volatile sulfur compound contents in breath [30].

*W. cibaria* produces lower levels of lactic acids, secretes water soluble glucan and hydrogen peroxide, and thereby prevents halitosis or detal caries [12,13]. In this work, *W. cibaria* CMU was isolated from saliva obtained from Korean adolescent with good oral health.

As shown in the above results, five probiotics reduced volatile sulfur compounds formed by *F. nucleatum* and *P. gingivalis*, and *W. cibaria* CMU showed the highest activity among them, suggesting that *W. cibaria* CMU may be used as an oral care probiotics.

## 4. Materials and Methods

### 4.1. Bacterial Strains and Growth Conditions

*Weissella cibaria* CMU (US 7250162B2, CMU), *Streptococcus salivarius*-1 (S. sal-1), *Streptococcus salivarius*-2 (S. sal-2), *Lactobacillus salivarius* (L. sal), *Lactobacillus reuteri* (L. reu) were used in this study. *W. cibaria* CMU was obtained from Oradentics Co., Ltd. (Seoul, South Korea), and *S. salivarius* (S. sal-1, S. sal-2) were isolated from commercial probiotic products using tryptic soy agar (TSA, Difco, Detroit, MI, USA). *L. salivarius* (L. sal) and *L. reuteri* (L. reu) were also isolated from commercial probiotics products using De Man, Rogosa, and Sharpe agar (MRS agar, Difco). All bacterial strains were identified through 16S rRNA sequence analysis. *S. mutans* Ingbritt, *S. sobrinus* B13, *F. nucleatum* ATCC 10953 and *P. gingivalis* ATCC33277 were provided by Chonnam National University.

*Streptococcus cultures* were grown in tryptic soy broth (TSB): *Weissella* and *Lactobacillus* cultures were grown aerobically, in MRS broth at 37 °C for 16 h. *F. nucleatum* cultures were grown in brain heart infusion broth (BHI broth, Difco) supplemented with 1% yeast extract (Difco), 0.1% cysteine (Sigma, St. Louis, MO, USA), 10 µm/mL hemin (Kisan Bio Co., Ltd., Seoul, South Korea), 5 µm/mL menadione (Kisan Bio Co., Ltd., Seoul, South Korea). *P. gingivalis* cultures were grown anaerobically in TSB supplemented with 0.5% yeast extract, 0.05% cysteine, 10 µm/mL hemin, and 5 µm/mL menadione at 37 °C for two days.

### 4.2. Lysozyme Resistance on Bacterial Growth

Growth inhibition potential of lysozyme (Sigma) for lactic acid bacteria was determined by monitoring survival ratio at TSA or MRS. The pellet was obtained by centrifugation of inoculum of 5 mL (OD_600_ = 10^9^ cells/mL) from 16 h cultures at 3500 rpm for 10 min at 4 °C PBS buffer (10 mL) containing 100 mg lysozyme/L was added to the pellet. The lysozyme treatments were incubated at 37 °C for 30 and 90 min, and the survival ratios of bacteria were measured by TSA or MRS agar cultures [31,32].

### 4.3. Hydrogen Peroxide Resistance on Bacterial Growth

The growth inhibition potential of hydrogen peroxide (Sigma) on lactic acid bacteria was determined by monitoring the survival ratio in TSA or MRS agar. An inoculum of 0.1 mL (OD_600_ = 5 × 10^8^ cells/mL) from overnight cultures was incubated in 10 mL of PBS buffer containing 1 mM H_2_O_2_ at 37 °C for 1, 3, and 6 h, and the survival ratios of bacteria were measured by TSA or MRS agar cultures [33].

### 4.4. Acidogenic Potential

To evaluate the acidogenic potential of lactic acid bacteria, *Weissella* and *Lactobacillus* were grown in MRS minimal medium (proteose peptone number 3, beef extract, polysorbate 80, ammnonium citrate, sodium acetate, MgSO_4_, MnSO_4_, dipotassium phosphate) supplemented with 4% glucose, 4% fructose, 4% lactose, 4% sucrose, or 1.5% yeast extract, while *Streptococcus* was grown in TSB minimal medium (pancreatic digest of casein, papaic digest of soybean, sodium chloride, dipotassium phosphate) with the same supplements used for *Weissella and Lactobacillus*. An inoculum of 0.1 mL (OD_600_ = 0.05) from overnight cultures were incubated aerobically at 37 °C for 24 h, and the pH and total microaerobes were measured. PAV (production of acid value) was calculated as follows [34]. PAV = pH × Log CFU/mL.

### 4.5. Hydrogen Peroxide Estimation

Lactic acid bacteria cultures were centrifuged at 3500 rpm for 4 min at 4 °C, the supernatant was then neutralized to pH 7.0 and filtered through a syringe (0.45 µm). The filtrates were assayed based on colorimetry using a hydrogen peroxide assay kit (ab102500, Abcam, Cambridge, MA, USA) [35]. The optical density was read at 570 nm by a spectrophotometer using 100 µL of supernatant placed in 96-well enzyme-linked immunosorbent assay (ELISA) microplate.

### 4.6. Inhibition of Biofilm Formation

*S. mutans* cultured at 37 °C overnight was adjusted to OD_600_ = 0.5 (~5 × 10^8^ CFU/mL) and diluted 10 times using TSB with 5% sucrose (TSB-S). Lactic acid bacteria cultured at 37 °C overnight were adjusted to OD_600_ = 0.5 (~5 × 10^7^ CFU/mL) and diluted 0.0625, 0.125, and 0.25 times using TSB-S or MRS broth with 5% sucrose(MRS-S). The *S. mutans* culture (0.1 mL; (~5 × 10^6^ CFU/mL) was inoculated on a 96 well plate, and then, serial dilution of lactic acid bacteria cultures (0.1 mL; (~5 × 10^6^ CFU/mL to ~6.25 × 10^5^ CFU/mL) were added to each well for inoculation with *S. mutans* (*S. mutans*:lactic acid bacteria = 1:1, 2:1, 4:1, 8:1))*.* After incubation at 37 °C for 24 h, media was removed from the wells, and plate wells were washed five times with sterilized distilled water. Plates were air dried for 10 min and each well was stained with 0.1 mL of 0.5% crystal violet solution in water for 15 min. After staining, plates were washed five times with sterilized water. The biofilm formed on the side of each well was dissolved in 99% ethanol and measured at 595 nm using a microplate reader (SpectraMax i3 Platform, Molecular Devices, Bismarckring, Austria) [36].

### 4.7. Antibacterial Activity

Lactic acid bacteria cultured aerobically at 37 °C for 16 h were centrifuged at 8000 rpm for 30 min and supernatants were filtered by syringe (0.45 µm). Samples were prepared using two, four, and eight times dilutions with TSB or growth medium for periodontal bacteria. Cultures of cariogenic or periodontopathic bacteria were adjusted to OD_600_ = 0.05 (~5 × 10^7^ CFU/mL) using each growth medium. Supernatants of lactic acid bacteria (0.1 mL) were inoculated on 96-well plates with cariogenic or periodontopathic bacteria (0.1 mL). After anaerobic incubation at 37 °C for 24 h. Each well was measured at 600 nm using a microplate reader (SpectraMax i3 Platform) [37].

### 4.8. Coaggregation Reaction

Cultures of bacteria were centrifuged at 3500 rpm for 10 min and the pellets obtained were adjusted to OD_600_ = 1 with Cisar’s buffer (1 mM Tris(hydroxymethyl) aminomethane (pH 8.0), 100 µM CaCl_2_, 100 µM MgCl_2_, and 0.15 M NaCl). Each bacteria or 1:1 mixture of periodontal and lactic acid bacteria was incubated at 37 °C in a shaking incubator (~110 rpm) for 30 min. After incubation, the cultures were left standing for more than 3 min before 0.5 mL of the supernatants were measured at 600 nm using a spectrophotometer (SpectraMax i3 Platform) [38]. The coaggregation was calculated as follows:

$$\mathrm{Coaggregation}\;\left(\%\right)=\frac{\left(Ax\;+\;Ay\right)/2\;-\;A\left(x\;+\;y\right)\;}{\left(Ax\;+\;Ay\right)/2}\times 100$$

where *x* and *y* represent each of the two strains in the control tubes, and (*x* + *y*) the mixture.

### 4.9. Inhibition of VSC (Volatile Sulfur Compounds) Production

Mixtures (1:1) of VSC-producing bacteria (0.1 mL) and lactic acid bacteria (0.1 mL), were each adjusted to OD_600_ = 0.5 (~5 × 10^8^ CFU/mL) and were cultured under anaerobic conditions in 15 mL tubes containing 1 mL of each growth medium at 37 °C for 24 h. A sample of the vapor above the cultures was removed using a gas-tight syringe, and VSC(H_2_S, CH_3_SH) production was measured via Oral Chroma (CHM-1, ABILIT, Osaka, Japan) [12].

The inhibition was calculated as follows:

$$\mathrm{Inhibition}\;\left(\%\right)=\frac{\mathrm{VSC}\;\mathrm{of}\;\mathrm{monoculture}\;-\;\mathrm{VSC}\;\mathrm{of}\;\mathrm{mixed}\;\mathrm{culture}}{\mathrm{VSC}\;\mathrm{of}\;\mathrm{monoculture}}\times 100$$

### 4.10. Statistics

Experiments were replicated three times, and SPSS ver. 12.0 (SPSS Inc., Chicago, IL, USA) was used for statistical analysis. One-way ANOVA was conducted for the significance test between groups, and Duncan’s multiple range test was used to determine significant differences between the mean values (*p* < 0.05).

## 5. Conclusions

Probiotics have different benefits in oral care. Among the five probiotics used in this study, *W. cibaria* CMU showed a strong survival rate under poor oral conditions, and several positive results, including antibacterial activities, production of less acid and more hydrogen peroxide, inhibition of biofilm formation and VSC production, and efficient coaggregation. These results suggest that *W. cibaria* CMU is applicable as a novel oral care probiotic. Further studies are needed to elucidate its potential for use in the treatment of oral diseases.

## Figures and Tables

**Table 1:** The ability of oral probiotic strains to produce acid.

Probiotics	PAV *					*F* Values
	Glu	Fru	Suc	Lac	YE	
CMU	37.29 ± 0.09 ^a^	40.09 ± 0.05 ^b^	47.28 ± 0.04 ^c^	41.71 ± 0.19 ^d^	56.31 ± 0.09 ^e^	15,055.652 ***
S. sal-1	26.08 ± 0.04 ^a^	26.44 ± 0.07 ^a^	28.58 ± 0.15 ^b^	28.74 ± 0.44 ^b^	51.03 ± 0.06 ^c^	7563.223 ***
S. sal-2	22.28 ± 0.03 ^a^	29.62 ± 0.15 ^b^	25.03 ± 0.04 ^c^	29.48 ± 0.01 ^d^	37.13 ± 0.04 ^e^	17,975.809 ***
L. sal	31.1 6± 0.08 ^a^	29.00 ± 0.01 ^b^	32.78 ± 0.05 ^c^	32.33 ± 0.15 ^d^	44.97 ± 0.01 ^e^	18,270.924 ***
L. reu	35.82± 0.19 ^a^	39.16 ± 0.01 ^b^	36.37 ± 0.01 ^c^	39.87 ± 0.10 ^d^	56.47 ± 0.15 ^e^	16,185.140 ***

* Production of acid value (PAV) is calculated by multiplying pH by Log CFU/mL. *** *p* < 0.001. Glu: glucose, Fru: fructose, Suc: sucrose, Lac: Lactose, YE: Yeast Extract. ^a–e^ Duncan’s multiple range comparison.

**Table 2:** Dose-dependent effects of oral probiotic strains on the formation of *S. mutans* biofilms expressed as inhibition level (%).

Probiotics	*S. mutans*:Probiotics			
	1:1	2:1	4:1	8:1
CMU	94.7 ± 0.3	96.0 ± 0.1	96.8 ± 0.5	95.4 ± 0.1
S. sal-1	59.5 ± 2.8	54.6 ± 3.4	51.5 ± 2.8	24.1 ± 2.0
S. sal-2	0	0	0	0
L. sal	93.9 ± 0.5	90.7 ± 2.3	84.6 ± 4.4	78.8 ± 4.5
L. reu	85.3 ± 4.0	0	0	0

**Table 3:** Dose-dependent effects of oral probiotic culture supernatants on the growth of cariogenic bacteria, *F. nucleatum* and *P. gingivalis*, expressed as inhibition level (%).

Probiotics	*F. nucleatum*				*P. gingivalis*			
	1:1	2:1	4:1	8:1	1:1	2:1	4:1	8:1
CMU	97.9 ± 0.1 ^a^	96.0 ± 0.5 ^a^	90.6 ± 0.8 ^a^	36.1 ± 2.8 ^a^	96.9 ± 0.4 ^a^	99.0 ± 0.2 ^a^	98.8 ± 0.2 ^a^	99.7 ± 0.3 ^a^
S. sal-1	41.3 ± 2.8 ^b^	12.9 ± 3.1 ^b^	3.9 ± 4.5 ^b^	0.0 ^b^	82.9 ± 2.7 ^b^	55.5 ± 7.4 ^b^	28.7 ± 5.9 ^b^	0.0 ^b^
S. sal-2	47.0 ± 1.2 ^c^	15.2 ± 8.5 ^b^	2.0 ± 6.2 ^b^	0.0 ^b^	80.0 ± 3.4 ^b^	36.5 ± 9.7 ^c^	9.9 ± 0.7 ^c^	5.2 ± 15.6 ^c^
L. sal	97.4 ± 0.3 ^a^	94.9 ± 0.4 ^a^	97.8 ± 0.4 ^c^	94.3 ± 0.8 ^c^	96.6 ± 0.5 ^a^	96.7 ± 0.6 ^a^	98.2 ± 0.2 ^a^	98.9 ± 0.1 ^a^
L. reu	97.3 ± 0.1 ^a^	95.7 ± 1.1 ^a^	97.0 ± 0.4 ^c^	51.3 ± 2.5 ^d^	95.7 ± 0.5 ^a^	97.4 ± 0.8 ^a^	98.1 ± 0.4 ^a^	98.5 ± 0.4 ^a^
*F* values	1389.452 ***	358.904 ***	795.049 ***	1551.685 ***	52.484 ***	84.464 ***	800.976 ***	607.859 ***

*** *p* < 0.001. ^a–d^ Duncan’s multiple range comparison.

**Table 4:** Dose-dependent effects of oral probiotic culture supernatants on the growth of periodontal bacteria, *S mutans* and *S. sobrinus*, expressed as inhibition level (%).

Probiotics	*F. nucleatum*				*P. gingivalis*			
	1:1	2:1	4:1	8:1	1:1	2:1	4:1	8:1
CMU	90.9 ± 3.4 ^a^	40.1 ± 8.1 ^a^	0.0 ^a^	0.0 ^a^	96.2 ± 0.3 ^a^	81.9 ± 5.1 ^a^	3.8 ± 20.2 ^a^	0.0
S. sal-1	42.8 ± 1.6 ^b^	22.8 ± 7.5 ^b^	15.3 ± 3.4 ^b^	11.1 ± 4.1 ^b^	35.5 ± 17.8 ^b^	12.0 ± 19.2 ^b^	0.0 ^a^	0.0
S. sal-2	40.4 ± 4.0 ^b^	19.6 ± 4.0 ^b^	13.2 ± 5.7 ^b^	13.4 ± 4.2 ^b^	37.1 ± 6.1 ^b^	0.0 ^c^	0.0 ^a^	0.0
L. sal	96.3 ± 0.2 ^c^	96.0 ± 1.9 ^c^	47.3 ± 12.5 ^c^	0.0 ^a^	96.3 ± 0.5 ^a^	96.3 ± 0.5 ^d^	91.6 ± 2.5 ^b^	0.0
L. reu	94.2 ± 1.3 ^a,c^	79.8 ± 4.5 ^d^	0.0 ^a^	0.0 ^a^	95.1 ± 0.5 ^a^	94.7 ± 0.4 ^a,d^	5.8 ± 8.0 ^a^	0.0
*F* values	380.033 ***	110.069 ***	27.888 ***	20.166 ***	45.148 ***	131.017 ***	173.838 ***	

*** *p* < 0.001. ^a–d^ Duncan’s multiple range comparison.

**Table 5:** Coaggregation of probiotic strains with cariogenic bacteria or periodontopathic bacteria.

Probiotics	Coaggregation (%)			
	*S. mutans*	*S. sobriuns*	*F. nucleatum*	*P. gingivalis*
CMU	5.8 ± 0.6 ^a^	0.0 ^a^	81.2 ± 0.4 ^a^	3.7 ± 1.2 ^a^
S. sal-1	0.0 ^b^	13.8 ± 1.3 ^b^	78.9 ± 0.3 ^b^	0.0 ^b^
S. sal-2	0.0 ^b^	2.5 ± 0.2 ^c^	72.7 ± 0.7 ^c^	1.5 ± 0.8 ^c^
L. sal	0.0 ^b^	0.0 ^a^	0.0 ^d^	0.0 ^b^
L. reu	0.0 ^b^	0.0 ^a^	49.6 ± 0.5 ^e^	0.0 ^b^
*F* values	328.737 ***	309.850 ***	16,212.931 ***	20.446 ***

*** *p* < 0.001. ^a–e^ Duncan’s multiple range comparison.

**Table 6:** Inhibitory effects of oral probiotic strains on the production of H_2_S and CH_3_SH by *F. nucleatum* and *P. gingivalis*.

Strains	VSC (ppb) by *F. nucleatum*			VSC (ppb) by *P. gingivalis*		
	H_2_S	CH_3_SH	Inhibition (%)	H_2_S	CH_3_SH	Inhibition (%)
Mono	25,640 ± 702	37,225 ± 782	0	12,791 ± 432	33,553 ± 876	0
CMU	1352 ± 1272 ^a^	527 ± 152 ^a^	97.0	2095 ± 182 ^a^	717 ± 171 ^a^	93.9
S. sal-1	3540 ± 835 ^b^	1532 ± 500 ^b^	91.9	1849 ± 172 ^a^	1423 ± 78 ^b^	92.9
S. sal-2	6777 ± 838 ^c^	2030 ± 246 ^b,c^	86.0	1531 ± 82 ^b^	1475 ± 210 ^b^	93.5
L. sal	7081 ± 290 ^c^	2278 ± 323 ^c^	85.1	1844 ± 78 ^a^	1335 ± 130 ^b^	93.1
L. reu	9470 ± 833 ^d^	2242 ± 505 ^c^	81.4	3253 ± 204 ^c^	2290 ± 162 ^c^	88.0
*F* values	40.340 ***	11.592 ***		57.013 ***	38.527 ***	

*** *p* < 0.001. ^a–d^ Duncan’s multiple range comparison. Volatile sulfur compounds (VSC) which include hydrogen sulfide (H_2_S) and methyl mercaptan (CH_3_SH).
